# Editorial Notes

**Published:** 1949-04

**Authors:** 


### Editorial Notes

**Ministerial Dicta.**

A FEW weeks ago the Minister of Health urged improvements in hospital out-patient departments, such as appointments and comfortable chairs in waiting-rooms. Appointments have been given for over twenty years. Before that, in the dark ages, the doctor would hand his patient a note, saying : " Take that to the hospital to-morrow at 10.30 ". Was it worse to wait in the out-patient department for an hour or even two (seldom more) or as now to wait five or six weeks for an appointment ?

Our standard of comfort is certainly modest. Yet the arrangements in our large teaching hospitals are at least as good as those in " private " (e.g. Workmen's Aid Association) hospitals, managed entirely by the patients' representatives. For years the problem for *all* management committees has been how to get money for essential treatment : they have had little to spare for anything beyond this. It was pleasant to feel that now at last it was possible to do many things that financial stringency has hitherto forbidden.

Any complacency on this score was very quickly dissipated by the order to reduce estimates by one-eighth or even one-sixth, coupled with the injunction that the interests of the patients are not to be sacrificed. In hospital expenditure salaries for nursing and domestic staff account for nearly two-thirds ; food and drugs for patients for more than half the balance ; maintenance and repair of buildings and equipment, administration and medical staff payments together about one-sixth. The problem is, therefore, almost insoluble. At least we can rejoice that the interests of the patients, the sole *raison d'être* of the hospitals, are still paramount : and not, as some had begun to suspect, the collection of elaborate and probably useless statistics.

